# Measuring Microtemporal Processes Underlying Preschoolers’ Screen Use and Behavioral Health: Protocol for the Tots and Tech Study

**DOI:** 10.2196/36240

**Published:** 2022-09-28

**Authors:** Layton Reesor-Oyer, Hannah Parker, Sarah Burkart, Michal T Smith, Roddrick Dugger, Lauren von Klinggraeff, R Glenn Weaver, Michael W Beets, Bridget Armstrong

**Affiliations:** 1 Department of Exercise Science University of South Carolina Columbia, SC United States

**Keywords:** ecological momentary assessment, accelerometery, objective screen time monitoring, mobile phone

## Abstract

**Background:**

Excessive screen time is associated with poor health and behavioral outcomes in children. However, research on screen time use has been hindered by methodological limitations, including retrospective reports of *usual* screen time and lack of momentary etiologic processes occurring within each day.

**Objective:**

This study is designed to assess the feasibility and utility of a comprehensive multibehavior protocol to measure the digital media use and screen time context among a racially and economically diverse sample of preschoolers and their families. This paper describes the recruitment, data collection, and analytical protocols for the Tots and Tech study.

**Methods:**

The Tots and Tech study is a longitudinal, observational study of 100 dyads: caregivers and their preschool-age children (aged 3-5 years). Both caregivers and children will wear an Axivity AX3 accelerometer (Axivity Ltd) for 30 days to assess their physical activity, sedentary behavior, and sleep. Caregivers will complete ecological momentary assessments (EMAs) for 1 week to measure child behavioral problems, caregiver stress, and child screen time.

**Results:**

The Tots and Tech study was funded in March 2020. This study maintains rolling recruitment, with each dyad on their own assessment schedule, depending on the time of enrollment. Enrollment was scheduled to take place between September 2020 and May 2022. We aim to enroll 100 caregiver-child dyads. The Tots and Tech outcome paper is expected to be published in 2022.

**Conclusions:**

The Tots and Tech study attempts to overcome previous methodological limitations by using objective measures of screen time, physical activity, sedentary behavior, and sleep behaviors with contextual factors measured by EMA. The results will be used to evaluate the feasibility and utility of a comprehensive multibehavior protocol using objective measures of mobile screen time and accelerometry in conjunction with EMA among caregiver-child dyads. Future observational and intervention studies will be able to use this study protocol to better measure screen time and its context.

**International Registered Report Identifier (IRRID):**

DERR1-10.2196/36240

## Introduction

### Background

Excessive screen time for children is linked with poor sleep, inactivity, and behavioral problems [1-4], and only few children meet the World Health Organization’s recommendation that children under 5 years receive ≤1 hour of screen time per day [5,6]. The ways in which families use screens have changed dramatically over the past 2 decades, in large part owing to the introduction of mobile and interactive digital media devices. Historically, children’s screen time comprised mostly television use, which took place mainly at home. With new forms of digital media such as mobile phones and tablets, families can now use screens across many different locations and contexts. The pervasive availability of mobile devices has resulted in a dramatic increase in children’s use of mobile media. Children’s mobile screen time increased from 5 minutes per day in 2011 to 55 minutes per day in 2020, with nearly half (46%) of the children aged 2 to 4 years and more than two-thirds (67%) of the children aged 5 to 8 years having their own mobile device (tablet or smartphone) [7]. Videos on the web now constitute two-thirds of children’s screen viewing (66%), supplanting traditional television, which now accounts for only 23% of the average daily video screen time [7]. Unfortunately, our understanding of mobile screen use and its impact on child health lags behind the accelerated adoption of mobile technology.

Measuring screen time is complicated and has historically relied upon time-consuming, expensive observational methods conducted by highly trained staff or upon parent recall survey methods [8-12]. In a 2021 systematic review of young (aged 0-6 years) children’s screen time, none of the 622 studies used an objective measure of screen time [13]. However, there was only a moderate correlation between self-reported and objectively observed media use (*r*=0.38). Of the 49 studies that examined adults’ digital media–use behaviors, only 3 were within 5% of their objectively measured mean [10]. Another important limitation of commonly used screen time measures is their provision of global estimates of screen use, which is often gathered from a single question that asks parents to estimate television use in a *typical* day [14,15]. This global measure of screen time precludes the ability to examine the context or timing of screen use within and across days, thus limiting the understanding of the complex and changing patterns of children’s daily screen use. Therefore, updated, objective, and low-burden measures of screen use and its context are needed.

Recent developments in the passive monitoring of mobile screen use have the potential to provide novel insights into how, when, where, and why children and families use digital media (ie, mobile phones and tablets) throughout the day [16-18]. This method of digital media monitoring is both less expensive than direct observation and less burdensome for participants than self-reporting measures [15]. Similarly, advances in ecological momentary assessments (EMAs) allow for the assessment of the context and timing of behaviors while minimizing recall bias. Finally, advances in accelerometry have made it possible to passively collect continuous 24-hour data on children’s sedentary behavior, physical activity, and sleep over extended periods (ie, 30 days). While these technologies have been tested in isolation [8,16,19,20], to date, no studies have leveraged multiple streams of data from passive mobile sensing, EMA, and accelerometry to specifically study young children’s screen time [13,21]. It is unclear whether the confluence of these methods can be successfully used to gather meaningful information about the digital media use of children and families. Furthermore, the emerging literature on passive mobile sensing has been conducted with high-income White families and may not be generalizable [8,16].

### Objective

**Figure 1:**
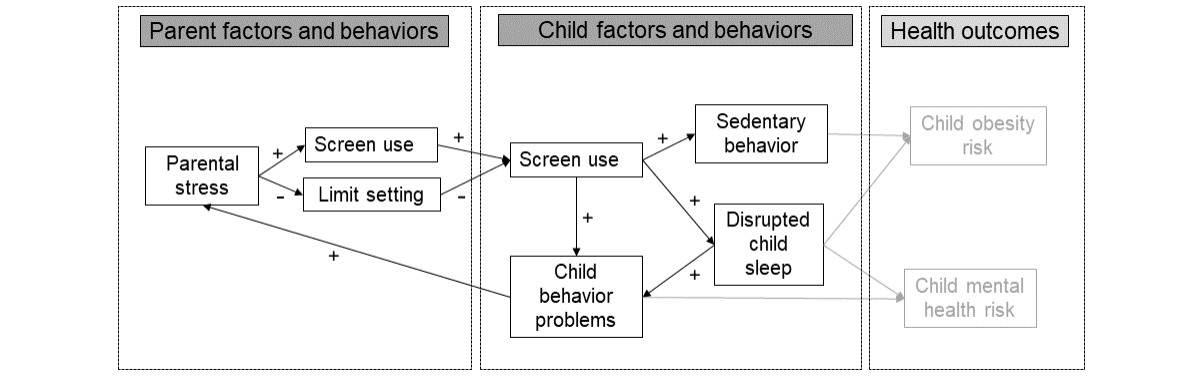
Conceptual model of proximal and distal associations among screen use, parent behaviors, and child behaviors.

We aim to evaluate the feasibility and utility of a comprehensive multibehavior protocol to measure digital media use and screen time context among a racially and economically diverse sample of preschoolers and their families. Figure 1 depicts the conceptual model of the associations between screen time context and children’s behaviors.

## Methods

### Design Overview

This study includes 100 caregiver-child dyads and uses a longitudinal, observational, dyadic, case-crossover design [22]. Using a case-crossover design allows a dyad to serve as its own control to assess the within-day effects of immediate antecedents on a dependent variable measured multiple times throughout the day and week [22]. Caregivers and their children aged 3 to 5 years will participate in a 30-day assessment protocol, with 50% (50/100) of the sample invited to participate in a second wave of data collection 3 to 12 months later to evaluate the feasibility of retaining the sample over time and to assess the longer-term acceptability of the protocol.

### Participants

Participants include racially and economically diverse caregivers and preschoolers in the greater Southeastern United States. The caregiver inclusion criteria are as follows: (1) being a primary caregiver of a child between the ages of 3 and 5 years, (2) owning a smartphone device, and (3) being able to read and speak English. The exclusion criteria for children include a diagnosis of a severe developmental or physical disorder that would prevent ambulation. This decision was made because of the inability to recruit a large enough sample of children to draw meaningful conclusions.

### Procedures

The study maintains rolling recruitment, with each dyad on their own assessment schedule, depending on the time of enrollment. Enrollment was scheduled to take place between September 2020 and May 2022.

We aim to recruit a nonrandom volunteer sample by posting fliers at daycare centers, pediatric clinics, and community centers such as food banks, as well as Facebook advertising in the form of “boosted” posts. To obtain a socioeconomically diverse sample, we will partner with daycares serving low-income families and prioritize the enrollment of low-income families. In addition, we will use snowball recruitment methods where participants are compensated for referring families who then successfully participate in the study.

Data collection is completely remote, in part driven by the necessary COVID-19 pandemic protocol adjustments [23]. Interested participants will be directed to an informational website through a QR code or hyperlink. The informational website describes the study procedures and research participant protections. The informational website includes a web-based consent form and directs interested participants to a short screener survey to assess initial study eligibility. A trained member of the research team will contact interested and eligible families by phone to answer any remaining questions and verbally confirm their desire to participate.

Following recruitment, the eligible child and caregiver dyads are texted a Qualtrics link to the baseline survey. The baseline questionnaire is designed to be completed within approximately 30 minutes. After caregivers complete the baseline survey, they are sent instructions to download a screen time monitoring app (Chronicle; Android devices) [18] or upload screenshots (iOS devices) depending on the make of their smartphone. Technical support is provided as required by the research team. Caregivers are then mailed 2 Axivity AX3 accelerometers (Axivity Ltd), one for themselves and one for their preschool-age child. Caregivers and their children are asked to wear the Axivity AX3 and monitor their screen time for 30 days. We will conduct a 30-day assessment to examine the higher limit of time that the families are willing to wear an activity watch and monitor their digital media use. The first week of the 30-day monitoring period includes 7 days of EMAs, which are texted to the primary caregivers’ smartphones. EMAs are limited to the first week (vs the entire monitoring period) to minimize participant burden, as this portion of the study requires active participation. The first week of the 30-day assessment was selected to allow for flexibility because of any potential issues that might prevent a participant from completing EMAs (eg, work conflicts). Participants who do not complete enough EMAs to earn a gift card during the first week (Compensation section) are offered an extension to complete additional EMAs. In line with the recent EMA studies [20], the Tots and Tech study allows flexibility in the survey completion window in an effort to retain families from diverse backgrounds who might experience additional barriers to completing measures within the time frame. Following the 30-day monitoring protocol, caregivers will complete a semistructured qualitative interview about their experiences. Participants who complete the study protocol (eg, do not drop out) in the first year (wave 1) are randomly selected (50/100, 50%) to be recontacted 3 to 12 months after the initial evaluation and invited to repeat the entire research protocol for additional compensation. Families that decline to participate in the second assessment are asked to complete a short web-based survey regarding their experiences and reasons for declining. The number of families that decline or are lost to follow-up is documented to assess protocol feasibility.

### Baseline Survey

#### Overview

The following information is assessed using survey measures administered through the Qualtrics platform. Caregivers report on the following sociodemographic variables for themselves and their child: birthday, biological sex, and race and ethnicity. Caregivers indicate their relationship with the child, marital status, employment status, and education, as well as the following household characteristics: number of children and adults in the home, family income, and use of government assistance (eg, Supplemental Nutrition Assistance Program or Special Supplemental Nutrition Program for Women Infants and Children, medical assistance, etc). *Poverty ratio* is calculated based on the caregiver’s report of family income and number of dependents using federal poverty thresholds that align with the year of data collection [24]. Caregivers complete the 2-item *food insecurity* screening questionnaire. This 2-item screener has shown excellent sensitivity and specificity (93% and 87%, respectively) with the 18-item US Department of Agriculture household food security scale [25], which is considered the gold standard for assessing food insecurity among children [26]. A 1-item measure is used to assess neighborhood safety, “How safe do you consider your neighborhood?” [27] Caregivers complete retrospective measures of chronic stress, including *perceived stress* in the past month (Perceived Stress Scale) [28] and confusion and *disorganization* in the home environment (CHAOS) [29], in addition to measures of *symptoms of depression and anxiety* (Center for Epidemiological Studies-Depression Scale and State-Trait Anxiety Inventory, respectively) [30,31]. Caregivers also complete measures assessing *child behaviors* (Strengths and Difficulties Questionnaire) [32] *and parenting satisfaction* [33]. Caregivers complete questionnaires regarding their child’s and their own *sleep habits* and routines [34-36] and *screen time* habits [37], function [38], and regulations [39], in addition to the number and types of screens in the home and in the child’s bedroom.

#### Height and Weight

Owing to COVID-19 pandemic restrictions [23], caregiver and child height and weight are collected via caregiver report on the baseline survey. BMI (kg/m^2^) is calculated for caregivers. As for children, age- and sex-specific BMI z-scores are determined using the Centers for Disease Control and Prevention criteria [40].

### EMA Surveys

Caregivers will complete the EMA surveys in the first week of the 30-day protocol. EMA surveys are sent to caregivers via SMS text messages to their smartphones. The SMS text messages contain a Qualtrics survey link to assess the domains using prompts listed in Table 1. Additional items that are only included in the end-of-day survey are listed in Table 2.

**Table 1:** Ecological momentary assessment (EMA) items.

Variable domain and item		Response options	Frequency or format
**Time spent with child**			
	How much time have you spent with your child in the past 2 h?	None <30 min 30-60 min 60-90 min 90-120 min	If “none,” skip to Stress Exposure questions
**Child mobile phone use**			
	In the last 2 h, how much has your child used your mobile phone?	None <30 min 30-60 min 60-90 min 90-120 min	Select one
**Child other screen time**			
	In the last 2 h, how much has your child watched television, played videogames, or used a computer?	None <30 min 30-60 min 60-90 min 90-120 min	If “none” on both Child Mobile Phone Use and Child Other Screen Time, skip Screen Function question
**Screen function [38]**			
	I let my child use these screens...	So that they could learn something As a reward for good behavior As they like it As part of a daily routine To allow myself free time	Select all that apply
**Child behavior problem intensity [41]**			
	In the last 2 h, how problematic has your child’s behavior been?	Not at all A little bit Moderate amount Quite a bit A great deal	Select one
**Child behavior problem content [42]**			
	Did your child lose their temper/have a temper tantrum in the last 2 h?	Yes No	Select one
	Did the tantrum last >5 min?	Yes No	If “yes” to the previous question
	Was this tantrum because they were...	Frustrated, angry, or upset Tired hungry or sick To get something she/he wanted Out of the blue	If “yes” to the previous question; select all that apply
	Did your child disobey or break the rules/say “no” when told to do something in the last 2 h?	Yes No	Select one
	Did your child disobey or break the rules/say “no” because they were...	Frustrated, angry, or upset Tired hungry or sick To get something she/he wanted Out of the blue	If “yes” to the previous question; select all that apply
	Did your child act aggressively in the last 2 h?	Yes No	Select one
	Did your child act aggressively because they were...	Frustrated, angry, or upset Tired hungry or sick To get something she/he wanted Out of the blue	If “yes” to the previous question; select all that apply
**Stress exposure [43]**			
	In the last 2 h, which of these things caused you stress?	Work Demands at home Family Tension with a coworker Tension with a partner Tension with your child Something else	Select all that apply
**Stress [44]**			
	How stressed are you feeling right now?	Not at all A little Quite a bit Extremely	Select one
**Perceived stress or self-efficacy [45]**			
	How certain do you feel that you can deal with all the things that you have to do RIGHT NOW?	Not at all A little Quite a bit Extremely	Select one
**Affect [46]**			
	How frustrated/angry are you feeling? How sad/depressed are you feeling? How happy are you feeling? How calm/relaxed are you feeling?	Not at all A little bit Moderate amount Quite a bit Extremely	Select one
**Physical context**			
	Where were you when you received this message?	Home (indoors) Home (outdoors) Work (indoors) Outdoors (not at home) Indoors (not at home) Car/bus/train Other (specify)	Select one

**Table 2:** Ecological momentary assessment (EMA) prompts for end of day.

Variable domain and item		Response options	Frequency or format
**Parent sleep quality [35]**			
	How would you rate YOUR sleep quality last night?	0 (terrible) to 5 (excellent)	Select one
**Child sleep quality [47]**			
	How would you rate your CHILD’S sleep quality last night?	0 (terrible) to 5 (excellent)	Select one
**Child behavior**			
	Thinking back on today, how often did your child misbehave in ways that were dangerous or unsafe?	Not at all Once More than once	Select one
	Thinking back on today, how often did your child act aggressively toward adults?	Not at all Once More than once	Select one
**Screen time limiting**			
	How much did you stick to your “usual” rules around screen time?	1 (not at all) to 5 (completely)	Select one
**Bedtime rules**			
	How much did you stick to your “usual” routines and rules around bedtime?	1 (not at all) to 5 (completely)	Select one
**Mobile screen time**			
	For how many hours did your child use your mobile phone today?	0-12+ h	Select one
**Interactive screen time**			
	How many hours did your child use a tablet or computer or play video games today?	0-12+ h	Select one
**Passive screen time**			
	How many hours did your child watch television, videos, movies (not on a mobile device) today?	0-12+ h	Select one
**Time spent with the child [41]**			
	Since waking up this morning, how many hours have you spent with your child in the same location?	0-12+ h	Select one
**Daycare**			
	How many hours did your child attend preschool/daycare today?	0-10+ h	Select one
**Parent illness**			
	Were you sick today?	Yes No	Select one
**Child illness**			
	Was your child sick today?	Yes No	Select one
**Accelerometer compliance-parent**			
	Did you wear the activity watch all day today?	Yes No	Select one
	If not, why?	Lost/cannot locate Strap broken Uncomfortable Other (specify)	If “no” to the previous question
**Accelerometer compliance-child**			
	Did your child wear the activity watch today?	Yes No	Select one
	If not, why?	Lost/cannot locate Strap broken Uncomfortable Other (specify)	If “no” to the previous question

Caregivers are informed that they will receive 4 “short surveys” per day between 8:30 AM and 9 PM and that they have 2 hours to complete each survey. For example, if a survey is sent at 8:30 AM, caregivers will have until 10:30 AM to complete the survey. After 2 hours, the links expire, and caregivers will no longer be able to access the survey. The schedule of assessments differs over the course of the 7 days and thus appears random to participants. The exact schedule is presented in Table 3. The timing protocol uses 4 signal-contingent prompts, including 1 end-of-day EMA message, which occurs at 9 PM every night. The EMA surveys are delivered in nonoverlapping time windows and refer to the previous 2 hours. This timing was selected based on previous research indicating that ≤5 prompts per day are acceptable to families [48].

**Table 3:** Ecological momentary assessment (EMA) schedule^a^.

Day	Time			
Monday	9:30 AM	1 PM	7 PM	9 PM
Tuesday	8:30 AM	2 PM	7:30 PM	9 PM
Wednesday	9:30 AM	2:30 PM	6 PM	9 PM
Thursday	9 AM	2 PM	7:30 PM	9 PM
Friday	10:30 AM	3:30 PM	7 PM	9 PM
Saturday	9:30 AM	3 PM	7 PM	9 PM
Sunday	10 AM	2 PM	6 PM	9 PM

^a^Participants will receive an SMS text message at these times prompting them to complete an ecological momentary assessment that must be completed within 2 hours.

In line with the recent guidelines for EMA use [49], caregivers are provided training on completing the EMAs and given a practice opportunity before the start of the assessment period. Training comprises 1 practice EMA and 1 practice end-of-day survey (with additional questions at the day level), and participants have the opportunity to seek clarity on the survey items and procedures before the assessment period.

First, caregivers report how much time they have spent with their child over the previous 2 hours. Caregivers who indicate that they have not been with their child are not presented with survey items regarding their child’s behaviors. EMA prompts regarding the frequency and intensity of child behavioral problems are based on EMA items previously tested among parents of young children [41]. Items regarding specific disruptive behavior are adapted from the Multidimensional Assessment of Preschool Disruptive Behavior [42]. Exposure to stressors is assessed using items adapted from the Daily Hassles Scale [43,50]. Degree of stress is assessed from a single item, “How stressed are you feeling right now?” Responses range from “not at all” to “extremely” [44]. Time Spent with Child and Limit Setting items are informed by existing protocols using EMA among parents [43]. Caregiver report of the overall screen time is assessed using questions adapted from the National Health and Nutrition Examination Survey screen time survey [51]. Daily assessment of screen time is presumed to improve recall bias compared with 30-day recall measures.

Several methodological considerations were made when designing the EMA protocol to balance the benefits of data richness with the drawbacks of potential participant burden and demand characteristics. Reduced-item EMA subscales are used instead of the full scales in order to limit survey fatigue. In addition, EMA surveys are prompted at seemingly random times within preset intervals (ie, a hybrid signal-interval contingent sampling schedule) to prevent anticipatory effects, such as pausing or changing current behavior in anticipation of a survey prompt at a known time [52]. Despite the use of repeated measures, reactivity is generally low with EMA procedures [53]. Furthermore, the combination of EMA with accelerometer data minimizes the weakness of using either instrument independently [54].

### Passive Mobile Sensing (Screen Time Monitoring)

#### iOS Devices

**Figure 2:**
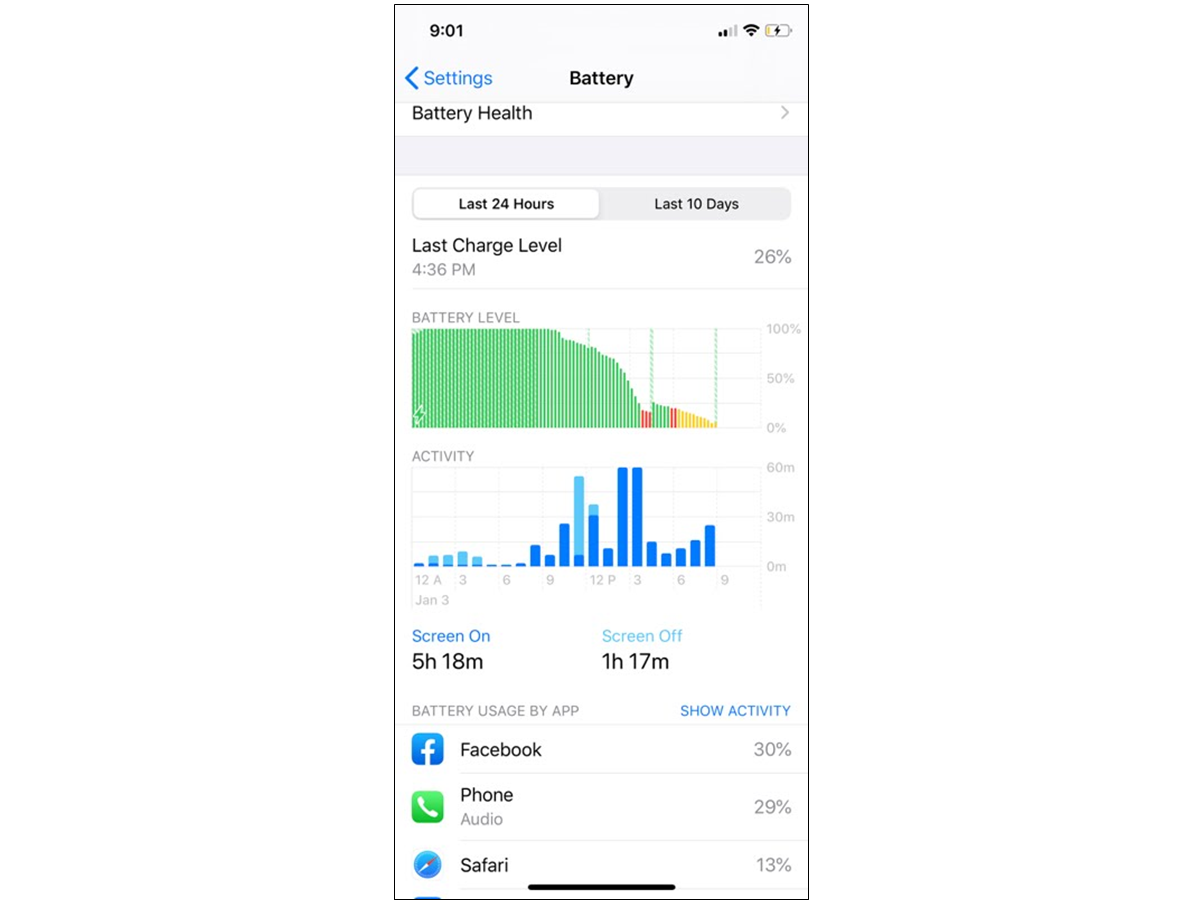
Example screenshot to objectively measure screen time on a participants’ iPhone.

Caregivers with an iPhone are texted with an automated reminder to send a screenshot of their screen time use each day at 9 PM to a study-specific phone number. The relevant information is located under the “Battery” tab in the iPhone settings. Figure 2 presents an example screenshot. Participants send the research staff 1 practice screenshot (those with iOS devices) before the start of data collection. Trained research staff verify that the iOS screenshot contains the correct information and provide personalized feedback to the participants as needed. If a caregiver fails to send a correct screenshot during the 7 days of EMAs, the study staff send a personalized SMS text message within 24 hours. After the 7-day EMA period, the study staff send a personalized SMS text message if a caregiver fails to send a screenshot on 2 consecutive days (48 h).

#### Android Devices

Caregivers with an Android device are provided instructions to enroll in Chronicle, an app designed specifically for passive screen time monitoring on Android devices [18]. Chronicle collects and transmits the timestamped app use data automatically to its platform. During the 30-day monitoring period, the research staff monitor the Chronicle dashboard daily to ensure regular uploading of the screen time monitoring data. If more than 48 hours has lapsed since the last data uploaded from an active participant, the research staff contact caregivers to troubleshoot and then contact Chronicle support staff as necessary. The use of technical support (participant use of research staff technical support, as well as research staff requests from Chronicle support staff) are recorded as a feasibility outcome.

As caregiver smartphones are monitored, we will use a coding scheme to identify which apps are likely to be used by caregivers versus their children [16]. Two independent coders will categorize each app as either a child or an adult app based on information from the Apple and Google Play app stores; a third coder will arbitrate disagreements. During the qualitative interviews, caregivers are queried about whether their most frequently used apps are primarily used by themselves or their child.

#### Child Devices

Caregivers whose children have a compatible device (iOS or Android) are invited to enroll their child’s device in the study using the same procedures as those used to enroll parents’ devices. However, child device enrollment is not a requirement for the study.

### Accelerometry

Both caregivers and children are asked to wear an Axivity AX3 (Axivity Ltd), a waterproof triaxial accelerometer, on their nondominant wrist for 30 days to assess physical activity, sedentary behavior, and sleep. The nondominant wrist placement improves compliance (compared with waist placement) [55]. Before data collection, the accelerometers are initialized using Open Movement (OMGui, version 1.0.0.43; Newcastle University) at a sample rate of 50 Hz with a range of +8*g* to −8*g*. Participants are mailed their accelerometers with reminders about wearing the device, a sticker chart for children to track the number of days worn, and a prestamped envelope to return the accelerometers following the 30-day protocol. The participants are instructed to wear the device at all times, including sleeping periods. In the event of a lost or damaged device, a replacement device is mailed to the participant. The number of lost or damaged devices is used to determine the utility and feasibility of deploying accelerometers over longer wear periods (ie, 30 days).

### Data Processing

Axivity data are downloaded using Open Movement (OMGui, version 1.0.0.43; Newcastle University). Raw Axivity AX3 .cwa accelerometer files are processed in R (R Foundation for Statistical Computing) using the GGIR package (version 2.6-4) [56]. During processing, GGIR autocalibrates the signal using local gravity as a reference, identifies abnormally high values, detects nonwear periods, and calculates the magnitude of the acceleration corrected for gravity. Files are excluded if the postcalibration error is >0.02*g* (a measure of acceleration) and if the wear time is less than 16 hours during the 24-hour period. The nonwear time is determined based on the SD and the value range of each accelerometer axes raw data during 15-minute blocks within a 60-minute window. These blocks are classified as nonwear time if the SD of the 60-minute window is <13 milli-g, and the value range of the 60-minute window is <50 milli-g for 2 of the 3 accelerometer axes [57]. The default method for nonwear time is used, as the average acceleration at similar times on days of the week is imputed for invalid data. We determine the time spent in physical activity intensity categories (eg, inactive, light, and moderate to vigorous) using the intensity thresholds (milli-g) described by Roscoe et al [58] and Hildebrand et al [59,60] for preschoolers and caregivers, respectively. Sleep outcomes are estimated using methods developed by van Hees et al [61], which identify periods of sustained inactivity when the z-angle does not change by >5° for at least 5 minutes.

### Qualitative Interview

Following the 30-day monitoring period, caregivers are contacted by phone to complete a semistructured interview with a member of the research team (Multimedia Appendix 1). The semistructured interview is designed to address the following constructs of the study protocol: relevance, comprehensiveness, comprehensibility, satisfaction, and frequency or rationale of nonadherence [62-65]. Feedback provided by caregivers during the semistructured interviews is used to inform protocol changes for the future iterations of the study.

### Compensation

In total, caregivers will receive up to US $180 in Amazon gift cards for participating in each wave of the study. Participants will be compensated with electronic gift cards that are delivered after they complete each study task. Caregivers will receive a US $15 gift card for completing the baseline survey. Caregivers who complete at least 21 EMA prompts (75%) will be considered compliant and receive a US $40 gift card. Participants who fail to complete 21 EMA prompts within the 7-day period will be given the option to extend their EMA period beyond 7 days to have additional survey opportunities. Those who complete 21 EMA prompts with additional days of surveys will be compensated the full amount (US $40). Caregivers will receive a US $30 gift card for completing at least 21 days (70%) of screen time monitoring on their smartphones. Caregivers will receive a US $50 gift card upon the return of the Axivity accelerometers; caregiver-child dyads who both wear the Axivity accelerometers for at least 21 valid days (70%) will receive an additional US $25 gift card. Finally, caregivers will be compensated US $20 for completing the semistructured phone interview.

### Wave 2

A total of 50% (50/100) of the families who complete the study protocol will be invited to participate in a second wave of data collection 3 to 12 months after their initial enrollment. The 9-month discrepancy in the timing of the second wave is due to rolling recruitment. The purpose of the second wave of data collection is to evaluate the feasibility of retaining the sample over time and to assess the longer-term acceptability of the protocol. The protocol will be deemed feasible and acceptable if 80% of those invited agree to re-enroll in wave 2 of the study. Furthermore, the wave 2 protocol will be adapted based on wave 1 participant feedback.

### Statistical Analyses

Data will be analyzed using IBM SPSS Statistics for Windows version 27. The descriptive statistics of the feasibility outcomes will be presented. As feasibility is the primary outcome of interest, we will use standard effect size estimates (ie, Cohen *d* and *r*) and minimal acceptable feasibility metrics in favor of significance testing [66]. Two-tailed independent sample *t* tests will be used to examine the differences between those who withdraw from the study and those who complete the protocol.

### Sample Size

A sample size of 100 is adequate to evaluate the feasibility of the multibehavior protocol to measure digital media use and screen time context among preschoolers and their families, allowing for generalization to other families with children of similar age and demographics. Given that this is a pilot study, no power analysis is required [67].

### Ethics Approval

The study protocol was approved by the institutional review board of the University of South Carolina (Approval Number: Pro00092634).

## Results

The Tots and Tech study was funded in March 2020. Data collection began in September 2020 and was completed in May 2022. We aim to enroll 100 caregiver-child dyads. The Tots and Tech outcome paper is expected to be published in 2022.

## Discussion

### Overview

Excessive screen time has been linked to poor physical health outcomes (ie, obesity) [68] and mental health outcomes (ie, attention-deficit/hyperactivity disorder and externalizing behaviors) [69,70]. However, studies evaluating the relationship between screen time and health behaviors have significant limitations. The existing studies on screen time have focused on television viewing, largely neglected digital media use [71], relied on retrospective parental reports of average screen time [12], and not yet examined individual variability or temporality [72] of screen time within a day. This study protocol is designed to address these weaknesses by (1) leveraging data already collected by smartphones and tablets (ie, app use) to objectively measure smartphone screen time and (2) integrating intensive longitudinal data collected from multiple sources (ie, passive mobile screen time sensing, EMA, and accelerometry).

This protocol attempts to overcome the methodological limitations of previous studies by incorporating objective measures of mobile screen time and health behaviors (physical activity, sedentary time, and sleep) in conjunction with EMA. The intention of this protocol is to measure screen time behaviors among caregivers and their preschool-age children more accurately. The results will be used to evaluate the feasibility and utility of a comprehensive multibehavior protocol to measure digital media use and screen time context. If this protocol is deemed feasible and acceptable, it can be used in future observational and intervention studies to better measure screen time and its context. In addition, this protocol makes it possible to examine more complex predictors of child health behaviors, such as the within-dyad daily dynamics and between-dyad associations between parenting practices and child behavior problems.

However, our methodology is not without limitations. Objectively monitoring mobile devices (eg, smartphones and tablets) can only provide objective information on digital media use, which is only one of the sources of screen time. It is likely that children and their caregivers also use other types of screen media (ie, television viewing, computers, and shared tablets). This additional screen use is assessed using a combination of EMA measures and traditional questionnaire data. Although the use of parent-report measures of children’s additional screen time is less than ideal, the EMA method of intermittent assessment could reduce the recall bias present in many existing measures of *typical* screen use. Using this EMA method of screen assessment can provide temporal context to examine the within-day variability of screen time patterns. Future studies could leverage the emerging advances in facial recognition and machine-learning technology to passively and objectively measure additional forms of screen use among families.

The current methods of smartphone-based digital media–use assessment are also limited by the inability to determine who is using the smartphone. We attempt to disentangle smartphone use by determining whether the apps used are designed for children or adults. During qualitative interviews, caregivers indicate whether frequently used apps are used by themselves or by their children and provide information about who has access to the smartphone during peak time windows.

For the purpose of this feasibility study, all device use is considered screen time. However, the protocol for monitoring screen time on Android (but not iOS) devices provides information on how the device is being used (eg, app use) at specific times throughout the measurement period. Future studies can use this protocol to evaluate the differential effects of different types of screen time. For example, do children exhibit more behavioral problems following playing games on their tablet compared to video chatting? These studies are needed to advance science and inform guidelines for children’s screen time.

To avoid overlapping time points and minimize missing data due to noncompliance [43], participants are sent 4 EMAs per day; these EMAs do not assess every hour of the day and, therefore, likely miss important screen time context and resultant child behaviors. Nevertheless, the methodology used by the Tots and Tech study greatly improves upon common methods in child screen time literature.

### Conclusions

This study is designed to evaluate the feasibility and utility of a comprehensive multibehavior protocol to measure digital media use and screen time context among a racially and economically diverse sample of preschoolers and their families. The findings will inform protocol adjustments in preparation for a future well-powered study. Ultimately, the Tots and Tech study aims to reveal the process-oriented science that underlies the association between screen time and physical (ie, sleep and activity) and mental health (ie, behavioral problems). Preliminary data from this study will inform model convergence statistics and provide informative priors for the intraclass correlation coefficient and path estimates. These are necessary to conduct a power analysis to inform a future well-powered observational cohort study to examine these connections in further detail over time. The study protocol uses EMAs and objectively measured methods that can reveal the temporal mechanisms of health behavior. Understanding individual-level behavioral patterns has the potential to advance the science of personalized intervention approaches and inform health behavior theories to improve the health and well-being of children and their families.

## Appendix

Multimedia Appendix 1 Tots and Tech qualitative interview guide.

## Abbreviations

EMA: ecological momentary assessment

## Data Availability

Data sharing is not applicable to this study, as no data sets were generated or analyzed during the study.
