# Supplementary material for: Measuring Microtemporal Processes Underlying Preschoolers’ Screen Use and Behavioral Health: Protocol for the Tots and Tech Study
Source: JMIR Res Protoc. 2022 Sep 28;11(9):e36240. doi: 10.2196/36240 (PMC9557980; doi:10.2196/36240)
Supplement: Multimedia Appendix 1 [file resprot_v11i9e36240_app1.docx]

Tots and Tech Qualitative Interview Guide

**Let’s begin by talking about the daily short surveys.**

1. Tell me about your experience completing the multiple daily short surveys?
   1. What worked well?
   2. Was there anything that you found challenging or difficult?
   3. What recommendations do you have for improving the experience completing the multiple daily short surveys?
2. I see that you responded to _ out of _ surveys. Thanks for doing that! For the survey’s you missed, what prevented you from completing them?
   1. Tell me what you thought about the length of the surveys.
   2. Tell me what you thought about the timing of when the surveys were sent.
3. In our study, you completed the short surveys for about 1 week. Would you be willing to complete the short surveys for a longer period of time? How long would you be willing to willing to complete the short surveys?
   1. Would you be willing to complete more surveys each day? How many short surveys would you be willing to complete each day?

**Now, let’s talk about the actual survey questions and what they asked.**

1. How well did the survey questions capture…
   1. Your stress? Can you think of other sources of stress we should ask about?
   2. Problem child behavior? What other problem behaviors should we include?
   3. Screen time? Were there any additional screen time behaviors that we should include?
   4. Were there any questions you would add or remove to better capture your screen time, child’s behavior, or stress?
   5. Were the answer choices for the short survey questions appropriate?
   6. Were there any answer choices you would add or remove to better capture your screen time, child’s behavior, or stress?
2. In the surveys we asked you to remember the past 2 hours – were there behaviors that we missed because of the timing??

**Let’s talk about the activity watches you and your child *(insert Child’s name)* wore.**

1. What did you think about wearing the activity watch? What did you think about having your child wear the watch?
   1. What worked well and what did you find challenging or difficult?
   2. What recommendations do you have for improving the experience?
   3. What did you think about wearing the watches **for 30 days**?
2. Was the watch comfortable to wear during the day/night? If not, how did you handle this?
   1. During times when your child did not wear the activity watch, why did they take it off?
   2. What strategies did you use to encourage your child to wear it?
   3. Would you and your child be willing to wear it again?

**Now I would like to learn more about your child’s screen time.**

1. Was your child’s screen time typical this month?
   1. How was it different this month? (if different)
   2. In your first survey, you said your child has access to the following screens: _____. Did that change this month?
   3. Was your phone use typical this month? If they say it was not typical: how was it different this month?
2. We found that ________ were the apps you used the most. Were these used by you, your child, or both?
   1. What are the apps your child uses?
   2. The time of day the phone was used most was ________ – tell me about what occurs during that time of day?
   3. Who besides yourself regularly uses your phone?

10.) You kept track of your screen time for a month, tell me about that experience.

1. What worked well?
2. Was there anything that you found challenging or difficult?
3. What recommendations do you have for improving the experience?
4. **iOS**: When you missed a screenshot, why did you miss it? What would have made it easier for you to complete all of the screenshots?
5. **Android:** Tell me about downloading the app for screen time monitoring and enrolling in the study. How easy/difficult was this?
6. Did you experience any technological issues with monitoring screen time?
7. Would you be willing to monitor your screen time again in the future?
8. Would you be willing to monitor your screen time for a longer period of time?
9. Would you be willing to let us monitor other types of screen time (computer, television, tablets) in a similar manner?

**The next questions are about ways we might ask families to keep track of screen time in future studies – not this study.**

- Would you be willing to monitor other screen media in your home? TV, videogame consoles? computer?
- Would you be willing to install software to let us monitor other screen media (e.g. TV, videogame, computer use)?
- Would you be willing to plug in a device that would passively monitor other screen media (e.g. TV, videogame, computer use)?
- What media would you not be comfortable monitoring? What might make you unwilling to monitor these things?
- There is some new technology that uses facial recognition to see if someone is looking at a screen. What would make you willing or unwilling participate in a research study that used this type of device?
- Many phone apps can use your location data. Would you be willing to share location data in a future research study? What would make you willing or unwilling to share location data?
- Would you be willing to wear a device that measures proximity (when you are close to your child)? What would make you willing or unwilling to wear this device?

**Let’s conclude by talking about compensation.**

1. What did you think about the gift card amount for completing the study?
2. What did you think about receiving $15 for completing the baseline survey?
3. What did you think about receiving $40 for completing the short surveys?
4. What did you think about receiving $75 for wearing the activity watches?
5. What did you think about receiving $30 for enrolling in the Screen Time Monitoring/ completing the screenshots?
6. Is there anything else you would like to tell us about your experience participating in the Tots & Tech Study?
7. Were there any additional drawbacks or benefits of participating in the study that you have not already mentioned?
8. Would you be willing to participate in the study again?
9. What motivated you to sign up/discontinue?
10. Why unwilling to participate? What parts? Would you be willing to do the other components of the study?
